# Impact of a Virtual Reality Intervention on Stigma, Empathy, and Attitudes Toward Patients With Psychotic Disorders Among Mental Health Care Professionals: Randomized Controlled Trial

**DOI:** 10.2196/66925

**Published:** 2025-01-21

**Authors:** Jing Ling Tay, Yuanrong Qu, Lucas Lim, Rohan Puthran, Chye Lee Robert Tan, Rajkirren Rajendran, Ker Chiah Wei, Huiting Xie, Kang Sim

**Affiliations:** 1 West Region Institute of Mental Health Singapore Singapore; 2 Department of Nursing Institute of Mental Health Singapore Singapore; 3 Department of Pharmacology National University of Singapore Singapore Singapore; 4 East Region Institute of Mental Health Singapore Singapore; 5 Case Management Unit Institute of Mental Health Singapore Singapore; 6 School of Health and Social Sciences Singapore Institute of Technology & University of Glasgow Singapore Singapore; 7 Yong Loo Lin School of Medicine National University of Singapore Singapore Singapore; 8 Lee Kong Chian School of Medicine Nanyang Technological University Singapore Singapore

**Keywords:** virtual reality, social distance, stigma, empathy, mental health, schizophrenia, psychosis, psychotic disorder, mental disorder, healthcare professional, VR, randomized controlled trial, RCT, user satisfaction

## Abstract

**Background:**

Previous studies have found that psychotic disorders are among the most stigmatized mental disorders. Of note, virtual reality (VR) interventions have been associated with improvements in attitudes and empathy and reduced stigma toward individuals with psychotic disorders, especially among undergraduates, but this has not been examined among mental health care professionals.

**Objective:**

We aimed to evaluate the effectiveness of a newly developed VR intervention for mental health care professionals to improve attitudes and empathy and reduce stigma toward people with psychotic disorders.

**Methods:**

We conducted a randomized controlled trial and recruited eligible mental health care professionals from a tertiary mental health care institution. Both arms (VR intervention and VR control groups) were evaluated at baseline, postintervention, and 1-month follow up. The evaluation included outcomes related to attitudes (modified attitudes toward people with schizophrenia scale), stigma (social distance scale, personal stigma scale), and empathy (empathetic concern subscale of the Interpersonal Reactivity Index). The experience with the VR intervention was assessed using a user satisfaction questionnaire, and qualitative feedback was gathered.

**Results:**

Overall, 180 mental health care professionals participated and completed the study. Both groups showed improvements in attitude, social distance, and stigma scores but not the empathy score following the intervention. The VR intervention group had better user satisfaction than the VR control group. In addition, certain outcome measures were positively associated with specific factors including female gender, higher education level, certain job roles, years of work, and presence of loved ones with a mental disorder.

**Conclusions:**

Both the intervention and control VR groups of mental health care professionals showed improvements in attitudes, stigma, and social distance toward people with psychotic disorders. Future longitudinal studies may want to evaluate the impact of VR on caregivers and the public on these same and other outcome measures to reduce stigma and improve empathy toward individuals with psychotic disorders.

**Trial Registration:**

clinicaltrials.gov NCT05982548; https://clinicaltrials.gov/study/NCT05982548

## Introduction

Psychotic disorders can affect the individual in several domains including physical health, social functioning, occupational functioning, and quality of life. Patients with psychotic disorders experience positive symptoms such as hallucinations and delusions, and although there are effective treatments for these conditions, help-seeking behaviors and access to treatment may be affected by the attitudes, stigma, and empathy of mental health care staff regarding such conditions [1]. Empathy is the ability to place oneself in the shoes of another so as to better understand the experiences of the other person cognitively and emotionally [2], which can contribute positively toward the quality of patient care and the overall well-being of health care professionals [3]. In addition, among people with mental disorders, empathy of the therapist reduces feelings of isolation, increases hopefulness, and facilitates better therapeutic engagement with individuals with mental disorders [2]. Of note, better attitudes and empathy of health care staff toward patients with psychotic disorders have been associated with better patient well-being, quality of patient care, and treatment outcomes [4-6].

In turn, attitude and empathy levels can also be influenced by the level of stigma [7]. Stigma has been commonly associated with psychotic disorders [8]. Some negative perceptions of individuals with psychotic disorders included inaccurate and pejorative labels such as “unpredictable,” “violent,” and “aggressive,” even by health care workers [9,10]. Thus, stigma potentially prevents individuals who experience psychotic disorders from seeking help, as they fear discrimination [1]. A recent systematic review of 38 studies found that psychotic disorders are among the most stigmatized mental disorders among mental health care professionals [11], although mental health care professionals tended to have lower stigma levels [12], which could be related to direct social contact [13]. In particular, associative stigma among mental health care professionals was associated with more depersonalization, more emotional exhaustion, and less job satisfaction for the mental health professionals and higher self-stigma among patients [14]. Overall, the inter-relationships between stigma level and relevant mental health care professional factors such as age, job role, education level, and length of service are still not entirely clear [11].

Virtual reality (VR) interventions have been increasingly adopted to enhance training and quality of health care including mental health care [15-18]. In contrast to traditional videos, VR interventions can fully immerse users in the context, thus allowing users to experience specific simulated scenarios more realistically. A recent review found that VR and augmented reality interventions showed promise for improving the knowledge and attitudes toward individuals with neurocognitive and psychotic disorders [19,20]. Specifically, VR interventions that allowed participants to experience the sensory and cognitive challenges that people with dementia endured resulted in improvements in attitude levels among medical and pharmacy students [21]. Other VR interventions with simulated hallucinations also resulted in improvements in undergraduates’ attitudes and empathy levels toward people with psychotic disorders [22,23], but their effects on stigma remain inconclusive [24,25].

To date, limited studies have examined the use of augmented or VR-based modalities to improve attitudes, stigma, and empathy toward people with psychotic disorders. Previous studies were mainly conducted in the west among undergraduates, had pre-post designs, and reported some positive outcomes [22,23,25]. To the best of our knowledge, no study has examined the impact of VR intervention on mental health care professionals’ attitudes, stigma, and empathy levels toward people with psychotic disorders. Thus, in this study, we sought to examine the use and impact of a VR intervention containing simulations of psychotic experiences for mental health care staff in the context of a non-western culture. Based on extant data, we hypothesized that it could improve attitudes, reduce stigma, and enhance empathy toward patients with psychotic disorders among mental health care professionals.

## Methods

### Ethical Considerations

The study was a 2-arm, randomized controlled trial and was approved by the Institutional Review Boards at the Institute of Mental Health (IRRC number: 818-2022) and National Healthcare Group (IRB number 2023/00027) in Singapore.

### VR Intervention Group

**Figure 1:**
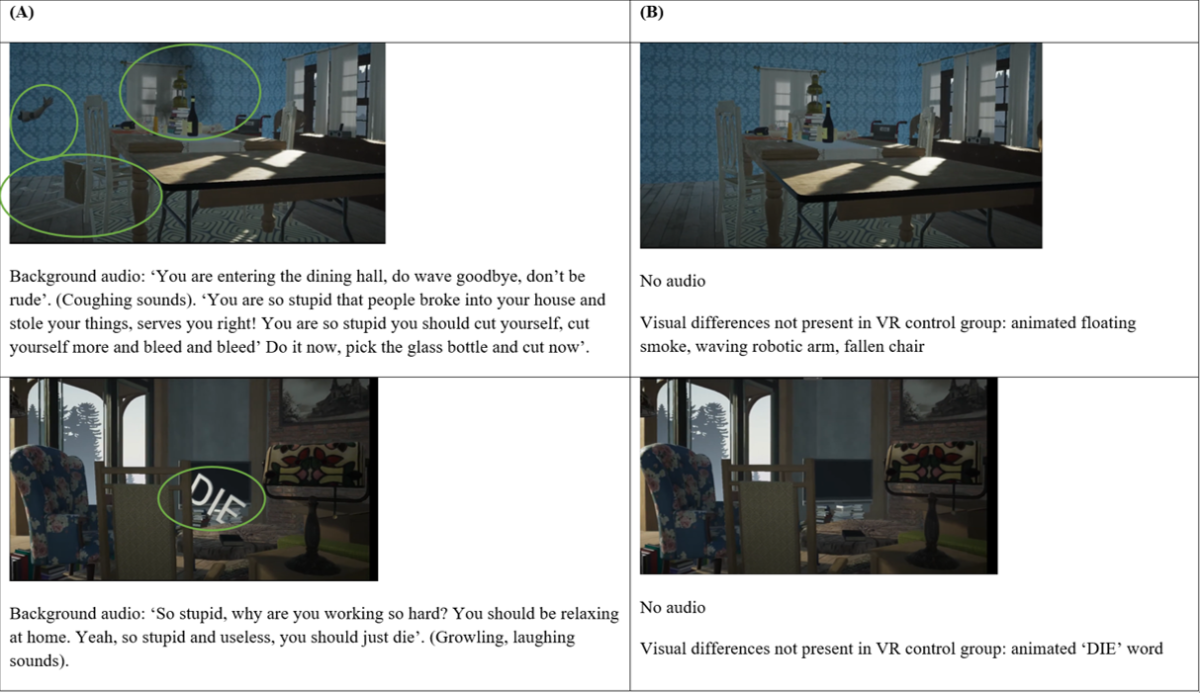
Examples of the display shown to the (A) intervention group and (B) control group.

The VR intervention was created using Unreal Engine [26]. The VR intervention illustrated a home scenario (Figure 1), and participants experienced simulated psychotic phenomena including auditory hallucinations. The constructed VR intervention is accessible at [27].

The VR intervention was constructed based on a systematic review conducted by the team [20] that found that effective interventions for reducing stigma and enhancing empathy and positive attitudes toward people with psychotic disorders included short VR videos with simulated visual and auditory hallucinations that lasted no more than 15 minutes [22,23]. The VR intervention was also based on existing simulation videos for psychotic disorders such as schizophrenia [28-31] that included common hallucinations including floating items; monsters or shadow-like figures; random negative words; auditory hallucinations including laughing, crying, or scary sounds; running commentary; and commands. The VR intervention was further revised following input from a clinician and a peer support specialist. The VR intervention, lasting no more than 7 minutes, was delivered in a single setting. In the process of obtaining consent, participants were informed that they might experience some discomfort during the VR intervention. They were informed that they could withdraw from the research study at any point in time. Each participant met a researcher within a quiet office room. They then participated in the VR intervention, which was conducted using a smartphone device inserted into a VR headset equivalent to Google Cardboard. Participants remained seated in a stationary position while they viewed the VR intervention. The intervention began with a house scene. Upon entering the house, the participant initially heard some voices. Later, the “voices” became increasingly frequent and emotionally charged with negative content as the scene went through different parts of the house. Participants would also experience “visual hallucinations” such as seeing the words “die” and “poison” in food and that corresponded with the “voices.” Eventually, the voices mentioned suicidal ideas, and the scene finally ended with a concerned call from a mental health care professional.

### VR Control Group

The VR control group viewed the scenario of the same home setting without the experience of the simulated visual and auditory hallucinations and the call by the mental health care professional. The constructed VR control intervention is accessible at [32].

### Participants

The participants were recruited from the only tertiary mental health institution in Singapore using stratified sampling according to their job role, namely (1) allied health staff (including occupational therapists, medical social workers, case managers), (2) nurse, or (3) physician.

#### Inclusion and Exclusion Criteria

The inclusion criterion entailed that the participant was involved in the direct care of patients with mental disorders. The exclusion criteria included (1) participants who were unable to use VR interventions due to reasons including motion sickness, disorientation, nausea, and vomiting or (2) those with a history of epilepsy, as flashing VR images might predispose these individuals to photosensitive seizures.

#### Sample Size Calculation

The sample size was calculated based on the effect sizes of earlier studies that evaluated the effectiveness of a VR intervention on positive attitudes toward people with mental disorders in Australia [22] and another that examined the effects of a VR game on stigma regarding mental disorders in Spain [33]. With an effect size of 0.48, power of 0.8, and probability level of 0.05 in detecting a difference, the estimated sample size was 70 participants per group [34]. Considering a dropout rate of 20% [35], the recommended sample size was determined to be 84, which was rounded to 90 per group, thus totaling 180 participants for both the intervention and control groups.

#### Recruitment

The participants were recruited through clinical, educational, and journal club meetings on virtual platforms such as Zoom and Teams as well as through emails and work messaging platforms. After confirmation of their interest in participating in the study, written informed consent was obtained by a research team member.

#### Randomization and Masking

Participants were then allocated to their relevant research group: the VR intervention group or the VR control group. Allocation used preprepared, concealed allocation letters and was conducted before the administration of the baseline questionnaires. An independent manager outside of the research team generated a random number list from a random number generator. The manager placed each random number within sealed and opaque envelopes. Upon recruitment, the researchers opened each sealed envelope to randomize each participant, thus ensuring blinding of the participants to the 2 separate arms.

### Data Collection Process

All questionnaires were completed via an online survey. One week after completion of the baseline questionnaires, the intervention group received the VR intervention, while the control group received a control VR intervention. Participants from both groups received a small reimbursement at this point. The second set of questionnaires was administered postintervention, and a final set of questionnaires was administered 1 month after the VR intervention. Participants from both groups were then compensated with another small reimbursement for their time and efforts following completion of the entire study. After completing the entire study, the control group was informed of their randomized arm and given access to the VR intervention.

### Data Collected Including Outcome Variables

#### Overview

Basic demographic data included age, gender, and nature and years of occupation. The primary outcomes were (1) attitudes, (2) stigma, and (3) empathy levels toward individuals experiencing psychotic disorders. Qualitative feedback regarding the VR intervention was also collected. The questions used in the questionnaire are listed in Multimedia Appendix 1.

#### Attitudes Toward People With Psychotic Disorders

Attitudes toward people with psychotic disorders were measured with the modified attitudes toward people with schizophrenia scale [36]. The 7-item scale is measured on a 9-point Likert scale ranging from 1 to 9. Total scores range from 7 to 63, with higher scores indicating better attitudes. Items 1 and 2 are reverse coded. An example item is “How much do you personally care about the plight of people with schizophrenia?” The internal consistency Cronbach alpha ranges from 0.76 to 0.79 [36].

#### Stigma

Stigma was measured using 2 tools: social distance scale and personal stigma scale. The social distance scale evaluates a participant’s willingness to be associated socially with someone who is experiencing a specific mental disorder [37-39]. The 8 questions are rated on a 4-point Likert scale: yes, definitely (0); yes, probably (1); probably not (2); and definitely not (3). The scores range from 0 to 24, with higher scores indicating greater social distance. Some examples of the questions include willingness to spend time, make friends, or be neighbors with someone with a mental disorder. In Australia, among 3021 young people aged 15 years to 25 years, an exploratory structural equation demonstrated that the desire for social distance scale achieved factor loading of 0.75 to 0.88. The modeling also demonstrated that the desire for social distance scale was distinct from perceived and personal stigma [40]. Among 3006 participants in Asia, factor loadings of the social distance scale ranged from 0.69 to 0.91 [41]. The scale also had a reliability Cronbach alpha of 0.85 [41].

The personal stigma scale consists of 2 subscales: dangerous/unpredictable and weak-not sick [40]. The questions are scored from 0 (strongly disagree) to 4 (strongly agree). Total scores range from 0 to 36, with higher scores indicating greater stigmatizing attitudes. Within Asia, the “weak-not sick” and “dangerous/unpredictable” subscales had reliability Cronbach alphas of 0.55 and 0.66, respectively [41]. For each scale, confirmatory factor analysis revealed a goodness-of-fit index of 0.94, Tucker–Lewis index of 0.96, and root mean square error of approximation of 0.08 for the personal stigma scale [41] and a goodness-of-fit index of 0.94, Tucker–Lewis index of 0.96, and root mean square error of approximation of 0.08 for the social distance scale [41].

#### Empathy

Empathy was measured with the empathic concern subscale of the Interpersonal Reactivity Index [42]. The 7-item subscale is measured on a 5-point Likert scale ranging from 0 (does not describe me well) to 4 (describes me very well). Total scores range from 0 to 28, with higher scores indicating greater empathy. Of the 7 items, 3 are reverse-coded. The scale achieved internal consistency Cronbach alphas of 0.68 to 0.79. Test-retest reliability (60-75 days) ranged from 0.61 to 0.81 [42].

#### Experience With the VR Intervention

The experience with the VR intervention was measured at postintervention with user satisfaction and motion sickness scores. First, user satisfaction was measured with a 6-item scale on a 7-point Likert scale ranging from 1 (strongly disagree) to 7 (strongly agree). Examples of questions were, “I benefit from the VR intervention” and “the VR intervention helps me to understand what people with schizophrenia experience.” In addition, there were 4 open-ended questions regarding feedback about the VR interventions to evaluate the participants’ perceptions of the intervention: (1) Please list the strengths of the intervention. (2) Please list how the intervention can be improved. (3) What did you enjoy most about the VR intervention? (4) What did you enjoy least about the VR intervention?

Second, the Visually Induced Motion Sickness Susceptibility Questionnaire [43] was used to measure motion sickness**.** It consists of 6 questions that evaluate the presence of adverse effects from using the VR intervention, with responses ranging from “never” (0) to “often” (3). The potential adverse effects include headache, nausea, giddiness, fatigue, and eyestrain. The scores of the scale range from 0 to 18, with higher scores indicating more adverse effects. The scale had a Cronbach alpha of 0.84, indicating good internal reliability.

### Data Analyses

An intention-to-treat analysis was conducted. All statistical analyses were performed using SPSS version 27 (IBM Corp) [44]. Data normality was evaluated with the Shapiro-Wilk test, skewness, kurtosis, Q-Q plots, and histograms [45]. Categorical variables were compared between the 2 groups using contingency tables (chi square), and continuous variables were compared using independent *t* tests. Correlation statistics were calculated to determine the relationship between demographic features and outcome measures. Repeated measures ANOVA was used to examine for time, group, and time × group differences. Assumption of sphericity was evaluated with the test of sphericity by Mauchly [46]. All *P* values were 2-tailed at the significance level of *P<.*05. The internal reliability of the scales was evaluated with the Cronbach alpha.

The open-ended questions about participant satisfaction with the intervention were analyzed using 6-step thematic analysis [47]. One author (JLT) perused the qualitative data to become familiar with it. The author then constructed a coding scheme related to the interview questions. The scheme included perceived strengths of the VR intervention and suggestions to improve it. It also included what participants found most or least useful about the VR intervention. The author evaluated the code to identify the initial themes, and additional analyses detailed each theme. All the codes and themes were subsequently reviewed independently by 3 other coauthors (YQ, HX, CLRT). Discrepancies were resolved through discussions among all the coauthors. Finally, verbatim comments were incorporated within the findings.

## Results

### Participants

**Figure 2:**
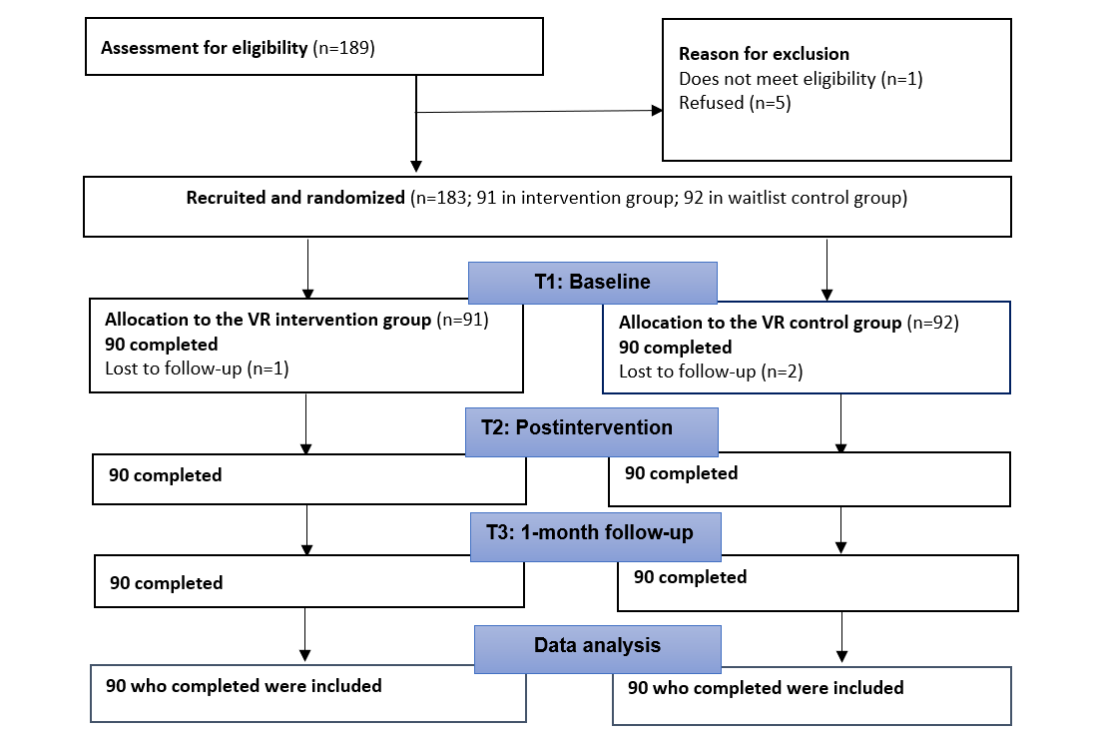
CONSORT (Consolidated Standards of Reporting Trials) diagram. VR: virtual reality.

Although 183 participants were originally recruited for the study from August 2023 through January 2024, 3 participants dropped out after randomization and did not complete any of the questionnaires. There were 30 participants in each job role (physician, nurse, and allied health) per arm, which totaled 180 participants for the 2 groups. All 180 participants completed the questionnaires at baseline, postintervention, and follow-up (see Figure 2 for the CONSORT [Consolidated Standards of Reporting Trials] diagram). Table 1 shows the demographic features and baseline rating scores of the participants. At baseline, there were no significant differences between the groups in demographic characteristics and levels of stigma, empathy, and attitude toward people with mental health disorders.

**Table 1:** Demographic features and rating scores for both virtual reality (VR) groups.

Characteristics and scores			VR intervention group (n=90)		VR control group (n=90)		Statistic (*df*)			*P* value	
**Gender, n (%)**								0.09 (1)^a^			.76
	Male	34 (38)		36 (40)							
	Female	56 (62)		54 (60)							
**Marital status, n (%)**								6.85 (5)^a^			.23
	Single, never married	31 (34)		18 (20)							
	Attached	16 (18)		18 (20)							
	Engaged	3 (3)		4 (4)							
	Married	39 (43)		49 (54)							
	Separated	1 (1)		0 (0)							
	Divorced	0 (0)		1 (1)							
**Highest education level, n (%)**								1.31 (4)^a^			.86
	“N” levels	0 (0)		1 (1)							
	Diploma	10 (11)		10 (11)							
	Basic degree	60 (67)		62 (69)							
	Masters	18 (20)		15 (17)							
	PhD	2 (2)		2 (2)							
**Occupation, n (%)**								1.33 (7)^a^			.99
	Psychiatrist	5 (6)		7 (8)							
	Psychiatry resident	10 (11)		13 (14)							
	Medical officer	13 (14)		10 (11)							
	Nurse	30 (33)		29 (32)							
	Medical social worker	6 (7)		6 (7)							
	Case manager	12 (13)		11 (12)							
	Occupational therapist	6 (7)		5 (6)							
	Others	8 (9)		9 (10)							
**Length of time working at the current hospital (years), n (%)**								5.92 (8)^a^			.66
	<1	14 (16)		17 (19)							
	1-3	24 (27)		25 (28)							
	4-6	21 (23)		15 (17)							
	7-9	9 (10)		7 (8)							
	10-15	15 (17)		18 (20)							
	16-20	4 (4)		1 (1)							
	21-24	2 (2)		5 (6)							
	25-30	0 (0)		1 (1)							
	>30	1 (1)		1 (1)							
**Overall length of time working in the mental health care sector (years), n (%)**								4.13 (8)^a^			.85
	<1	10 (11)		13 (14)							
	1-3	24 (27)		24 (27)							
	4-6	21 (23)		19 (21)							
	7-9	12 (13)		6 (7)							
	10-15	14 (16)		16 (18)							
	16-20	5 (6)		5 (6)							
	21-24	3 (3)		5 (6)							
	25-30	0 (0)		1 (1)							
	>30	1 (1)		1 (1)							
**Had a close friend or family with a mental disorder, n (%)**								1.81 (1)^a^			.18
	Yes	52 (58)		43 (48)							
	No	38 (42)		47 (52)							
Age (years), mean (SD)			33.40 (8.85)		33.56 (7.62)		0.14 (1, 178)^b^			.71	
Attitude score, mean (SD)			48.90 (5.75)		49.52 (5.12)		0.59 (1, 178)^b^			.44	
Social distance score, mean (SD)			8.27 (3.85)		7.98 (3.26)		0.30 (1, 178)^b^			.59	
Personal stigma score, mean (SD)			11.33 (4.37)		11.37 (4.23)		0.003 (1, 178)^b^			.96	
Empathy score, mean (SD)			20.24 (4.57)		20.07 (3.97)		0.08 (1, 178)^b^			.78	

^a^Chi-square test.

^b^*F* statistic.

### Attitudes Toward Individuals With Psychotic Disorders

#### Main Findings

The main time effect on attitudes was statistically significant (*F*_1.88, 335.35_=6.46, *P=.*002; Table 2). Post hoc analysis with Bonferroni adjustment found significant differences in attitude scores between baseline and postintervention (1.17, 95% CI –2.02 to –0.32; *P=.*003) and between postintervention and follow-up (–0.72, 95% CI 0.03 to 1.41; *P=.*04). The effects of (1) group and (2) the group × time interaction on attitudes were not significant.

**Table 2:** Comparisons of outcome measures across time using repeated measures ANOVA.

Outcome measure		Type III sum of squares (*df*)	*F* (*df*)	*P* value	ηp^2^
**Attitudes**					
	Group	1.90 (1)	0.02 (1, 178)	.88	<0.001
	Time points	125.89 (1.88)	6.46 (1, 539)	.002	0.04
	Group×time	47.72 (1.88)	2.45 (1, 538)	.09	0.01
**Social distance**					
	Group	6.02 (1)	0.16 (1, 178)	.69	0.001
	Time points	23.98 (1.87)	4.32 (1, 539)	.02	0.02
	Group×time	0.74 (1.87)	0.13 (1, 538)	.86	0.001
**Personal stigma**					
	Group	3.11 (1)	0.07 (1, 178)	.79	<0.001
	Time points	84.28 (2)	7.60 (1, 539)	.001	0.04
	Group×time	2.55 (2)	0.23 (1, 538)	.80	0.001
**Empathy**					
	Group	6.23 (1)	0.11 (1, 178)	.74	0.001
	Time points	17.92 (2)	2.31 (1, 539)	.10	0.01
	Group×time	10.80 (2)	1.40 (1, 538)	.25	0.008

#### Relationship Between Attitudes and Demographic Variables

Participants with loved ones with mental disorders had better attitudes than participants without (r_pb_=–0.16, *P=.*03). Better attitudes toward individuals with psychotic disorders were associated with a higher education level (ρ=0.2, *P=.*007), longer work experience durations in the current hospital and mental health sector (both ρ=0.16, *P=.*04), and a job role as a psychiatrist (r_pb_=0.15, *P=.*04; Table 3).

**Table 3:** Correlations between demographic features and outcome measures.

Participants’ characteristics		Attitudes		Social distance		Stigma			Empathy	
		Correlation	*P* value	Correlation	*P* value	Correlation	*P* value	Correlation		*P* value
Gender		0.12^a^	.12	–0.10^a^	.19	–0.02^a^	.76	0.20^a^		.008
Loved ones with a mental health condition		–0.16^a^	.03	0.18^a^	.01	0.14^a^	.06	–0.07^a^		.34
**Marital status**										
	Single, never married	–0.03^a^	.73	–0.003^a^	.96	0.005^a^	.94	–0.10^a^		.20
	Attached	–0.09^a^	.25	–0.02^a^	.78	–0.12^a^	.10	–0.06^a^		.44
	Engaged	–0.07^a^	.34	0.15^a^	.049	–0.07^a^	.35	0.02^a^		.79
	Married	0.13^a^	.08	–0.06^a^	.46	0.08^a^	.26	0.12^a^		.11
	Separated	0.03^a^	.74	0.02^a^	.81	0.12^a^	.12	–0.06^a^		.46
	Divorced	–0.11^a^	.13	0.10^a^	.17	0.12^a^	.12	0.07^a^		.37
**Occupation**										
	Psychiatrist	0.15^a^	.04	–0.009^a^	.90	–0.08^a^	.29	–0.03^a^		.73
	Resident	0.03^a^	.65	–0.14^a^	.07	–0.30^a^	<.001	0.08^a^		.31
	Medical officer	0.03^a^	.74	0.04^a^	.57	–0.08^a^	.27	0.15^a^		.04
	Nurse	–0.09^a^	.25	0.05^a^	.51	0.39^a^	<.001	–0.02^a^		.82
	Medical social worker	–0.03^a^	.72	–0.09^a^	.26	–0.15^a^	.04	0.04^a^		.62
	Case manager	–0.05^a^	.49	0.17^a^	.02	0.13^a^	.08	–0.10^a^		.17
	Occupational therapist	–0.01^a^	.85	–0.01^a^	.91	–0.09^a^	.25	–0.06^a^		.40
	Others	0.03^a^	.66	–0.09^a^	.25	–0.07^a^	.35	–0.07^a^		.35
Highest education level		0.20^b^	.007	–0.03^b^	.72	–0.24^b^	.001	0.05^b^		.49
Length of time working at the current hospital		0.16^b^	.04	0.09^b^	.24	0.18^b^	.02	0.07^b^		.35
Overall length of time working in the mental health care sector		0.16^b^	.04	0.08^b^	.30	0.15^b^	.05	0.07^b^		.35
Age		0.10^c^	.17	0.04^c^	.57	0.14^c^	.07	0.05^c^		.53

^a^Point biserial correlation.

^b^Spearman rank correlation coefficient.

^c^Pearson correlation.

### Social Distance

#### Main Findings

The main time effect on social distance was significant (*F*_1.87, 332_=4.32, *P=.*02; Table 2). Post hoc analysis with Bonferroni adjustment found a significant difference in social distance scores between baseline and follow-up (0.50, 95% CI 0.05 to 0.95; *P=.*02). The effects of (1) group and (2) the group × time interaction on social distance scores were not significant.

#### Relationship Between Social Distance and Demographic Variables

Participants without loved ones with a mental health condition had higher social distance scores (r_pb_=0.18, *P=.*01). In addition, higher social distance scores were associated with a job role as a case manager (r_pb_=0.17, *P=.*02) and being engaged (marital status; r_pb_=0.15, *P=.*049; Table 3).

### Stigma

#### Main Findings

The main time effect on stigma was significant (*F*_2, 356_=7.60, *P=.*001; Table 2). Post hoc analysis with Bonferroni adjustment found a significant difference in stigma scores between baseline and follow-up (0.97, 95% CI 0.34 to 1.60; *P<.*001). The effects of (1) group and (2) the group × time interaction on social distance scores were not significant.

#### Relationship Between Stigma and Demographic Variables

Lower stigma scores were associated with a higher education level (ρ=–0.24, *P=.*001) and job roles as a psychiatry resident (r_pb_=–0.30, *P<.*001) and medical social worker (r_pb_=–0.15, *P=.*04), and higher stigma scores were associated with longer work durations (ρ=0.18, *P=.*02) and a job role as a nurse (r_pb_=0.39, *P<.*001; Table 3).

### Empathy Level

#### Main Findings

The effects of time, group, and the group × time interaction on empathy levels were not significant.

#### Relationship Between Empathy Level and Demographic Variables

Greater empathy was associated with the female gender (r_pb_=0.20, *P=.*008) and a job role as a medical officer (Table 3).

### Experience With the VR Intervention (User Satisfaction and Motion Sickness Score)

The VR intervention group scored significantly higher (mean 33.18, SD 5.82; n=90) on the user satisfaction scale than the VR control group (mean 22.88, SD 9.35; n=90; t_149_=8.87, *P<.*001; Table 4). The motion sickness score was not significantly different between the 2 groups.

**Table 4:** Between-group comparisons of the outcome measures at the different time points.

Outcome measure		Intervention, mean (SD)	Control, mean (SD)	Mean difference (95% CI)	*t* (*df*)	*P* value	Cohen *d*
**Attitudes**							
	Pre-intervention	48.90 (5.75)	49.52 (5.12)	–0.62 (–2.22 to 0.98)	–0.77 (178)	.44	–0.11
	Postintervention	50.80 (6.28)	49.97 (5.42)	0.83 (–0.89 to 2.56)	0.95 (178)	.34	0.14
	Follow-up	49.73 (6.14)	49.59 (5.53)	0.14 (– 1.58 to 1.86)	0.17 (178)	.87	0.03
**Social distance**							
	Pre-intervention	8.27 (3.85)	7.98 (3.26)	0.29 (– 0.76 to 1.34)	0.54 (178)	.59	0.08
	Postintervention	7.88 (4.13)	7.64 (3.70)	0.23 (– 0.92 to 1.39)	0.40 (178)	.69	0.06
	Follow-up	7.68 (3.87)	7.57 (3.93)	0.11 (– 1.04 to 1.26)	0.19 (178)	.85	0.03
**Personal stigma**							
	Pre-intervention	11.33 (4.37)	11.37 (4.23)	–0.03 (– 1.30 to 1.23)	–0.05 (178)	.96	–0.008
	Postintervention	10.79 (3.82)	10.87 (4.60)	–0.08 (– 1.32 to 1.17)	–0.12 (178)	.90	–0.02
	Follow-up	10.21 (4.09)	10.56 (4.64)	–0.34 (– 1.63 to 0.94)	–0.53 (178)	.60	–0.08
**Empathy**							
	Pre-intervention	20.24 (4.57)	20.07 (3.97)	0.18 (– 1.08 to 1.44)	0.28 (178)	.78	0.04
	Postintervention	20.42 (4.63)	20.77 (4.39)	–0.34 (– 1.67 to 0.98)	–0.51 (178)	.61	–0.08
	Follow-up	20.07 (5.16)	20.54 (4.76)	–0.48 (– 1.94 to 0.98)	–0.65 (178)	.52	–0.10
User satisfaction postintervention		33.18 (5.82)	22.88 (9.35)	10.30 (8.01 to 12.59)	8.87 (178)	<.001	1.32
Motion sickness^a^ postintervention		3.64 (4.02)	3.56 (3.73)	0.09 (– 1.05 to 1.23)	0.15 (178)	.88	0.02

^a^Visually induced motion sickness susceptibility.

### Qualitative Findings

Thematic analysis of the data from the VR intervention group revealed 3 main themes: entering the patient’s world, benefits of VR learning, and boosting the VR intervention.

#### Theme 1: Entering the Patient’s World

Participants appreciated the experience of psychotic symptoms provided by the VR intervention, an experience that was novel, enlightening, and continuously described as “immersive” and “realistic.” Participants shared that “(It) gave me a perspective I would not have otherwise experienced” (male allied health professional, 1-3 years of work experience) and “It felt real, with voices talking to me constantly” (female nurse, 1-3 years of work experience).

The VR intervention allowed the participants to understand how hallucinations could affect the patient’s feelings and behaviors. One participant shared the following:

VR provided me with a chance to experience what auditory hallucinations can be like and also how it can impact our mood/actions even though it was just something as simple as walking through your house.

female allied health professional, 1-3 years of work experience

The effect of the intervention on evoking certain negative feelings was also apparent from participants’ feedback: “experience the agony that someone with continuous voices (is) going through” (female nurse, 10-15 years of work experience), “it triggered my emotions and made me feel annoyed at the voices” (male physician, 4-6 years of work experience), and “it was very real and very disturbingly hurtful” (female physician, 1-3 years of work experience). Hence, the VR intervention allowed the participants to better understand the nature of hallucinations as experienced by the patients, as well as the emotional and psychological distress accompanying them.

The experience allowed participants to experience not only the psychotic features and negative emotions but also their debilitating effects on orientation, state of mind, and overall functioning of the individual undergoing such experiences, as one participant illuminated:

Depiction of different forms of auditory hallucinations...visual hallucinations...unkempt state of the house...Overall, the hallucinations were appropriately frightening/disorienting.

male physician, 4-6 years of work experience

#### Theme 2: Benefits of VR

Participants described the use of VR as “new,” “refreshing,” “interesting,” “engaging,” “multisensory,” and “insightful,” which allowed them to better appreciate the subjective encounter of a psychotic disorder. Of note, participants stated that the intervention was an “innovative and immersive way to allow people to understand the experiences of patients with psychosis” (female physician, <1 year of work experience) and “very cool!” (female physician, <1 year of work experience).

Participants also commented on the duration, sound effects, and visuals effects of the VR intervention, for example, “The VR is professionally done” (male physician, 1-3 years of work experience). Other participants also appreciated the cost effectiveness, utility, ease of accessibility, and dissemination of the VR intervention. For example, one participant highlighted that “VR is so easily accessible with a pair of goggles and phone screen so it can be easily provided in the community setting” (female physician, 1-3 years of work experience).

#### Theme 3: Boosting the VR Intervention

Participants provided useful suggestions to enhance the VR intervention. First, participants suggested ways to make the intervention even more realistic, such as using locally accented voices, rather than the AI voices that came across as artificial, monotonous, and emotionless. They also recommended that the setting of the VR should be more reflective of the local context. Second, participants suggested ways to improve the comfort level when donning the gadgets. For example, participants commented that the headsets were slightly heavy or inappropriately sized. Third, a few participants requested narration to provide clarity on “some context of the person/environment” that they were about to experience (female allied health professional, 1-3 years of work experience). A number of participants requested that the VR intervention be more interactive, while other participants suggested highlighting the impact of these psychotic features, as one participant advised:

It would be interesting to see how the auditory hallucinations may have an impact on someone doing their daily tasks such as travelling, in work, or (in) social settings with friends.

female allied health professional, 1-3 years of work experience

### Discussion

### Principal Findings

There were several findings in this study. First, both groups showed improvements in attitudes, social distance, and stigma scores but not empathy following the VR intervention. Second, the VR intervention group had better user satisfaction than the VR control group. Third, the improvements in outcome measures were associated with specific factors including gender, education level, job role, years of work, and presence of loved ones with a mental disorder.

Both the VR intervention and VR control groups showed improvements in outcomes related to attitude, social distance, and stigma levels. These findings were congruent with those of earlier studies that found improved positive attitudes and reduced stigma toward people with mental disorders following evaluated interventions including immersive VR conditions related to schizophrenia [22,23,25,48,49], and dementia [21,50-52]. The specific element that rendered the VR intervention effective was its ability to allow participants to experience distressing psychopathology such as psychotic symptoms; this could lead to greater concern for the patients, which translates into better attitudes toward them [53]. Among the 5 earlier studies that evaluated a VR intervention for schizophrenia, only 2 had control groups [23,48]. Although the study by Marques et al [48] in Portugal evaluated outcomes of 2 arms (VR versus 2D video), the study by Kalyanaraman et al [23] in the United States evaluated the outcomes of 3 arms, namely, journaling of psychotic experiences, VR intervention alone, and VR intervention with journaling. For the latter study [23], the arm with journaling and VR intervention achieved the best outcomes in terms of better empathy, better attitudes, and reduced social distance, whereas the VR only intervention did not significantly perform better than the other arms. The VR only intervention performed the worst among all groups in terms of social distance. Another study also found that an intervention involving a simulation increased social distance [54]; this could be due to the fact that the experience during the VR intervention provoked negative emotional responses.

An earlier review of the use of simulated hallucinations to reduce stigma reported possible outcomes of negative affect and contradictory effect on stigma and cautioned about its use [24]. However subsequent VR studies reported improvement in stigma in several contexts, namely (1) a schizophrenia VR context whereby participants experienced schizophrenia symptoms while playing a character [55]; (2) the Inclúyete-VR platform, which includes components of role play in which the involved character participates in 6 interactive levels within a crisis center with exposure to psychosocial interventions aimed at recovery [56]; and (3) the Stigma-Stop platform, which presents characters with psychotic disorders [33] as well as other mental disorders such as dementia [57] and mixed anxiety-depression [35]. Among the earlier studies that evaluated VR interventions in schizophrenia, 2 had control group [55,56]. In both studies, the VR intervention performed better than patient review [55] and a neutral VR program [56] in terms of stigma outcomes. There were several reasons why both the VR intervention and VR control groups showed improvements in attitudes, social distance, and stigma. First, the participants in this study were mental health care professionals, who had direct, regular contact with individuals with psychotic disorders in their daily work, which facilitated improvements in attitudes and stigma toward people with psychotic disorders [13,58]. Second, the control VR intervention in this study, even without the added simulations of psychotic experiences, could be effectively immersive and realistic. Third, social desirability bias in both groups and positive expectation bias among the control VR participants could also have contributed to the positive outcomes observed within the groups [59]. Fourth, improvements in outcomes such as stigma could be influenced by concurrent educational or awareness events related to mental health and psychotic conditions that occurred within or outside the health care setting.

In this study, both groups did not show significant improvements in empathy scores postintervention or at the follow-up. This may be explained by the ceiling effect for empathy levels within mental health care staff [60,61] and the fact that empathy levels take time to improve [62]. Our findings differed from previous studies that found enhanced empathy levels after participation in interventions such as (1) a VR scenario in which the participants experienced schizophrenia symptoms [22,23,25,55]; (2) immersive VR interventions in dementia [51,57]; and (3) VR interventions surrounding psychosis, mood, and anxiety disorders [49]. Of note, none of these studies examined empathy scores longitudinally [22,25,49,51,57]. However, Slater et al [63], in their quasiexperimental study that evaluated a simulated dementia intervention (involving sensory and cognitive distortions while completing simple tasks such as folding apparel), found improvements at postintervention and the 3-month follow-up.

We found that the VR intervention group had significantly better user satisfaction than the VR control group, suggesting that the participants in the VR intervention group readily adapted to the VR scenario. This was supported by the qualitative feedback from the participants, who found the VR intervention realistic, engaging, and immersive by allowing them to experience psychotic symptoms. They also found that it evoked personal responses such as feeling the agony experienced by someone undergoing psychotic experiences. This was congruent with earlier studies in which participants of VR interventions for mental disorder [49] and dementia [51,52] gave positive feedback such as “interactive” and “interesting” and expressed appreciation for the opportunity to “experience the patient’s situation, thoughts, and feelings.”

There were several associations between the outcome measures and specific demographic and personal characteristics including female gender, higher education level, certain job roles, years of work, and presence of loved ones with mental disorder. We found that women had higher empathy scores, which was consistent with earlier studies [64]. One study specifically found that women had greater cognitive and emotional empathy than men [65], and biological and social factors may contribute to this association [66-69]. Congruent with extant literature [41,70,71], we found that a higher education level was associated with better attitudes and lower stigma regarding mental disorders, which may be related to better access to literature about mental disorders and the ability to understand the descriptions of mental disorders [72]. Longer durations of work experience were associated with better attitudes but higher stigma regarding mental disorders. We also found that, although certain occupational groups had more positive attitudes and better empathy scores (eg. psychiatrists) and lower levels of stigma (eg, residents and medical social workers), case managers and nurses had greater social distance and levels of stigma, respectively. This could be related to the higher workload amid demanding schedules for case managers and nurses within their work units, which can render them vulnerable to compassion fatigue and burnout [73]. In addition, we found that participants with loved ones with mental disorders had better attitudes and lower social distance levels, findings that agreed with the existing literature [73,74], highlighting the value of personal knowledge and care for someone with lived experience to lower stigma for mental disorders.

### Limitations and Future Directions

There were several limitations. First, the use of self-report measurements predisposed the study to social desirability bias among the participants. Second, researchers were not blinded due to the nature of the VR intervention, but data collection was conducted mainly online to reduce direct involvement of the researchers. Although participants were blinded, it was possible for participants to guess their eventual grouping. Third, the follow-up was only conducted for 1 month after the VR intervention. Fourth, the study did not include another comparison arm without VR. The effects of a non-VR control group on the outcome measures would allow us to better understand whether the positive changes were purely related to the use of VR or other underlying confounding variables related to the individual or context. Hence, future studies should include an additional arm without a VR intervention to observe for similar improvements or otherwise. The study can also be extended to other relevant groups such as families and caregivers of affected persons with psychotic disorders and the public to evaluate the impact on the various outcome measures. Further studies can examine the effects of the VR intervention on various types of stigma, such as patient self-stigma, professional stigma within mental health professionals, and social stigma among the public [75]. Longitudinal studies will be useful to examine the effectiveness and sustainability of VR interventions over a longer time period.

### Conclusion

In conclusion, both groups of mental health care workers participated in VR interventions and showed improvements in the outcomes related to attitudes, social distance, and level of stigma toward people with psychotic disorders. Future longitudinal studies may want to evaluate the impact of VR on these same and other outcome measures for caregivers and the public to reduce stigma and improve empathy toward individuals with psychotic disorders.

## Data Availability

Deidentified data can be requested via email to tay.jing.ling@aic.sg.

## Appendix

Multimedia Appendix 1 Online questionnaires.

Multimedia Appendix 2 CONSORT checklist.

## Abbreviations

CONSORT: Consolidated Standards of Reporting Trials
VR: virtual reality

## References

[1] Staiger T, Waldmann T, Oexle N, Wigand M, Rüsch Nicolas (2018). Intersections of discrimination due to unemployment and mental health problems: the role of double stigma for job- and help-seeking behaviors. Soc Psychiatry Psychiatr Epidemiol.

[2] Moudatsou M, Stavropoulou A, Philalithis A, Koukouli S (2020). The role of empathy in health and social care professionals. Healthcare (Basel).

[3] Ekman E, Krasner M (2016). Empathy in medicine: Neuroscience, education and challenges. Medical Teacher.

[4] Thornicroft G (2008). Stigma and discrimination limit access to mental health care. Epidemiol Psichiatr Soc.

[5] Holmqvist R (2000). Associations between staff feelings toward patients and treatment outcome at psychiatric treatment homes. J Nerv Ment Dis.

[6] Derksen F, Bensing J, Lagro-Janssen A (2013). Effectiveness of empathy in general practice: a systematic review. Br J Gen Pract.

[7] Dalky HF, Abu-Hassan HH, Dalky AF, Al-Delaimy W (2020). Assessment of mental health stigma components of mental health knowledge, attitudes and behaviors among Jordanian healthcare providers. Community Ment Health J.

[8] Rössler W (2016). The stigma of mental disorders: A millennia-long history of social exclusion and prejudices. EMBO Rep.

[9] Koschorke M, Oexle N, Ouali U, Cherian AV, Deepika V, Mendon GB, Gurung D, Kondratova L, Muller M, Lanfredi M, Lasalvia A, Bodrogi A, Nyulászi Anna, Tomasini M, El Chammay R, Abi Hana R, Zgueb Y, Nacef F, Heim E, Aeschlimann A, Souraya S, Milenova M, van Ginneken N, Thornicroft G, Kohrt BA (2021). Perspectives of healthcare providers, service users, and family members about mental illness stigma in primary care settings: A multi-site qualitative study of seven countries in Africa, Asia, and Europe. PLoS One.

[10] Linney C, Ye S, Redwood S, Mohamed A, Farah A, Biddle L, Crawley E (2020). "Crazy person is crazy person. It doesn't differentiate": an exploration into Somali views of mental health and access to healthcare in an established UK Somali community. Int J Equity Health.

[11] Valery K, Prouteau A (2020). Schizophrenia stigma in mental health professionals and associated factors: A systematic review. Psychiatry Res.

[12] Carrara BS, Ventura CAA, Bobbili SJ, Jacobina OMP, Khenti A, Mendes IAC (2019). Stigma in health professionals towards people with mental illness: An integrative review. Arch Psychiatr Nurs.

[13] Lien Y, Lin H, Lien Y, Tsai C, Wu T, Li H, Tu Y (2021). Challenging mental illness stigma in healthcare professionals and students: a systematic review and network meta-analysis. Psychol Health.

[14] Verhaeghe M, Bracke P (2012). Associative stigma among mental health professionals: implications for professional and service user well-being. J Health Soc Behav.

[15] Pottle J (2019). Virtual reality and the transformation of medical education. Future Healthc J.

[16] Torous J, Bucci S, Bell IH, Kessing LV, Faurholt-Jepsen Maria, Whelan P, Carvalho AF, Keshavan M, Linardon J, Firth J (2021). The growing field of digital psychiatry: current evidence and the future of apps, social media, chatbots, and virtual reality. World Psychiatry.

[17] Kuehn BM (2018). Virtual and augmented reality put a twist on medical education. JAMA.

[18] Choi J, Thompson CE, Choi J, Waddill CB, Choi S (2021). Effectiveness of immersive virtual reality in nursing education. Nurse Educ.

[19] Jones C, Jones D, Moro C (2021). Use of virtual and augmented reality-based interventions in health education to improve dementia knowledge and attitudes: an integrative review. BMJ Open.

[20] Tay JL, Xie H, Sim K (2023). Effectiveness of augmented and virtual reality-based interventions in improving knowledge, attitudes, empathy and stigma regarding people with mental illnesses-a scoping review. J Pers Med.

[21] Gilmartin-Thomas JF, McNeil J, Powell A, Malone DT, Wolfe R, Larson IC, O’Reilly CL, Kirkpatrick CM, Kipen E, Petrovich T, Bell JS (2018). Impact of a virtual dementia experience on medical and pharmacy students’ knowledge and attitudes toward people with dementia: a controlled study. JAD.

[22] Formosa NJ, Morrison BW, Hill G, Stone D (2020). Testing the efficacy of a virtual reality‐based simulation in enhancing users’ knowledge, attitudes, and empathy relating to psychosis. Australian Journal of Psychology.

[23] Kalyanaraman SS, Penn DL, Ivory JD, Judge A (2010). The virtual doppelganger: effects of a virtual reality simulator on perceptions of schizophrenia. J Nerv Ment Dis.

[24] Ando S, Clement S, Barley EA, Thornicroft G (2011). The simulation of hallucinations to reduce the stigma of schizophrenia: a systematic review. Schizophr Res.

[25] Silva RDDC, Albuquerque SGC, Muniz ADV, Filho PPR, Ribeiro S, Pinheiro PR, Albuquerque VHC (2017). Reducing the schizophrenia stigma: a new approach based on augmented reality. Comput Intell Neurosci.

[26] Unreal Engine.

[27] Kimo T Intervention fp 1 Unreal Editor 2022 06 27 00 27 27. YouTube.

[28] Drop Rate 'Schizophrenia Simulation' (Itchio) Full Playthrough. YouTube.

[29] lakerzz8 (2008). "Virtual Hallucinations", a virtual reality simulation of sc. YouTube.

[30] VVR Group (2020). VR Schizophrenia Simulation Full Demo. YouTube.

[31] Cohen E (2016). Schizophrenia: A Broken Mind 360°. YouTube.

[32] Kimo T Game firstperson 1 Unreal Editor 2022 05 04 00 31 23 without blood. YouTube.

[33] Cangas AJ, Navarro N, Parra JMA, Ojeda JJ, Cangas D, Piedra JA, Gallego J (2017). Stigma-Stop: a serious game against the stigma toward mental health in educational settings. Front Psychol.

[34] Sample size calculator. AI-Therapy Statistics.

[35] Yuen ASY, Mak WWS (2021). The effects of immersive virtual reality in reducing public stigma of mental illness in the university population of Hong Kong: randomized controlled trial. J Med Internet Res.

[36] Batson CD, Polycarpou MP, Harmon-Jones E, Imhoff HJ, Mitchener E C, Bednar L L, Klein T R, Highberger L (1997). Empathy and attitudes: can feeling for a member of a stigmatized group improve feelings toward the group?. J Pers Soc Psychol.

[37] Link BG, Cullen FT, Frank J, Wozniak JF (1987). The social rejection of former mental patients: understanding why labels matter. American Journal of Sociology.

[38] Bogardus ES (1933). A social distance scale. Sociology & Social Research.

[39] Social Distance Scale. Effective Services.

[40] Yap MBH, Mackinnon A, Reavley N, Jorm AF (2014). The measurement properties of stigmatizing attitudes towards mental disorders: results from two community surveys. Int J Methods Psychiatr Res.

[41] Subramaniam M, Abdin E, Picco L, Pang S, Shafie S, Vaingankar JA, Kwok KW, Verma K, Chong SA (2016). Stigma towards people with mental disorders and its components – a perspective from multi-ethnic Singapore. Epidemiol Psychiatr Sci.

[42] Davis MH (1980). A multidimensional approach to individual differences in empathy. JSAS Catalog of Selected Documents in Psychology.

[43] Golding JF, Rafiq A, Keshavarz B (2021). Predicting individual susceptibility to visually induced motion sickness by questionnaire. Front. Virtual Real.

[44] SPSS Statistics. IBM.

[45] Rani Das K, Rahmatullah Imon AHM (2016). A brief review of tests for normality. American Journal of Theoretical and Applied Statistics.

[46] Mauchly JW (1940). Significance test for sphericity of a normal n-variate distribution. Ann. Math. Statist.

[47] Braun V, Clarke V (2006). Using thematic analysis in psychology. Qualitative Research in Psychology.

[48] Marques AJ, Gomes Veloso P, Araújo M, de Almeida RS, Correia A, Pereira J, Queiros C, Pimenta R, Pereira AS, Silva CF (2022). Impact of a virtual reality-based simulation on empathy and attitudes toward schizophrenia. Front Psychol.

[49] Ho Yan Lam A, Jingxia Lin J, Wai Hin Wan A, Yuen Ha Wong J (2020). Enhancing empathy and positive attitude among nursing undergraduates via an in-class virtual reality-based simulation relating to mental illness. Journal of Nursing Education and Practice.

[50] Campbell D, Lugger S, Sigler GS, Turkelson C (2021). Increasing awareness, sensitivity, and empathy for Alzheimer's dementia patients using simulation. Nurse Educ Today.

[51] Wijma EM, Veerbeek MA, Prins M, Pot AM, Willemse BM (2018). A virtual reality intervention to improve the understanding and empathy for people with dementia in informal caregivers: results of a pilot study. Aging Ment Health.

[52] Ueno K, Tanaka H, Niki K, Ueda M, Tanaka A, Yokoi K, Naito Y, Ishii R (2023). Effects of real-time VR clinical practice on reducing the stigma toward dementia among students of occupational therapy: A randomized controlled trial. PCN Rep.

[53] Lara F, Rueda J (2021). Virtual reality not for "being someone" but for "being in someone else's shoes": avoiding misconceptions in empathy enhancement. Front Psychol.

[54] Brown SA, Evans Y, Espenschade K, O'Connor Maureen (2010). An examination of two brief stigma reduction strategies: filmed personal contact and hallucination simulations. Community Ment Health J.

[55] Zare-Bidaki M, Ehteshampour A, Reisaliakbarighomi M, Mazinani R, Khodaie Ardakani MR, Mirabzadeh A, Alikhani R, Noroozi M, Momeni F, Samani AD, Mehrabi Tavana MM, Esmaeili A, Mousavi SB (2022). Evaluating the effects of experiencing virtual reality simulation of psychosis on mental illness stigma, empathy, and knowledge in medical students. Front Psychiatry.

[56] Rodríguez-Rivas ME, Cangas AJ, Martin A, Romo J, Pérez JC, Valdebenito S, Cariola L, Onetto J, Hernández B, Ceric F, Cea P, Corrigan P (2024). Reducing stigma toward people with serious mental illness through a virtual reality intervention: a randomized controlled trial. Games Health J.

[57] Papadopoulos C, Kenning G, Bennett J, Kuchelmeister V, Ginnivan N, Neidorf M (2021). A visit with Viv: Empathising with a digital human character embodying the lived experiences of dementia. Dementia (London).

[58] Yamaguchi S, Mino Y, Uddin S (2011). Strategies and future attempts to reduce stigmatization and increase awareness of mental health problems among young people: a narrative review of educational interventions. Psychiatry Clin Neurosci.

[59] Williams JB, Popp D, Kobak KA, Detke MJ (2012). P-640 - The power of expectation bias. European Psychiatry.

[60] Matthews H, Williamson I (2016). Caught between compassion and control: exploring the challenges associated with inpatient adolescent mental healthcare in an independent hospital. J Adv Nurs.

[61] Gill L, Schaddelee M, Ramsey P, Turner S, Naylor T (2018). When empathy works: towards finding effective ways of sustaining empathy flow. Asia-Pacific Management and Business Application.

[62] Cameron CD, Hutcherson CA, Ferguson AM, Scheffer JA, Hadjiandreou E, Inzlicht M (2019). Empathy is hard work: People choose to avoid empathy because of its cognitive costs. J Exp Psychol Gen.

[63] Slater P, Hasson F, Moore K, Sharkey F (2021). Simulated based dementia training: impact on empathic understanding and behaviour among professionals and carers. Clinical Simulation in Nursing.

[64] Maximiano-Barreto MA, Fabrício DM, Luchesi BM, Chagas MHN (2020). Factors associated with levels of empathy among students and professionals in the health field: a systematic review. Trends Psychiatry Psychother.

[65] Mestre MV, Samper P, Frías MD, Tur AM (2009). Are women more empathetic than men? A longitudinal study in adolescence. Span J Psychol.

[66] Löffler CS, Greitemeyer T (2021). Are women the more empathetic gender? The effects of gender role expectations. Curr Psychol.

[67] Kamas L, Preston A (2021). Empathy, gender, and prosocial behavior. Journal of Behavioral and Experimental Economics.

[68] Rochat MJ (2023). Sex and gender differences in the development of empathy. J Neurosci Res.

[69] Andersen FA, Johansen AB, Søndergaard Jens, Andersen CM, Assing Hvidt E (2020). Revisiting the trajectory of medical students' empathy, and impact of gender, specialty preferences and nationality: a systematic review. BMC Med Educ.

[70] Holman D, Johnson S, O'Connor E, Diener E, Oishi S, Tay L (2018). Stress management interventions: Improving subjective psychological well-being in the workplace. Handbook of well-being.

[71] Crisp A, Gelder M, Goddard E, Meltzer H (2005). Stigmatization of people with mental illnesses: a follow-up study within the Changing Minds campaign of the Royal College of Psychiatrists. World Psychiatry.

[72] Girma E, Tesfaye M, Froeschl G, Möller-Leimkühler Anne Maria, Müller Norbert, Dehning S (2013). Public stigma against people with mental illness in the Gilgel Gibe Field Research Center (GGFRC) in Southwest Ethiopia. PLoS One.

[73] Corrigan PW, Nieweglowski K (2019). How does familiarity impact the stigma of mental illness?. Clin Psychol Rev.

[74] Lyndon AE, Crowe A, Wuensch KL, McCammon SL, Davis KB (2019). College students' stigmatization of people with mental illness: familiarity, implicit person theory, and attribution. J Ment Health.

[75] Subu MA, Wati DF, Netrida N, Priscilla V, Dias JM, Abraham MS, Slewa-Younan S, Al-Yateem N (2021). Types of stigma experienced by patients with mental illness and mental health nurses in Indonesia: a qualitative content analysis. Int J Ment Health Syst.

