# Supplementary material for: Impact of a Virtual Reality Intervention on Stigma, Empathy, and Attitudes Toward Patients With Psychotic Disorders Among Mental Health Care Professionals: Randomized Controlled Trial
Source: JMIR Ment Health. 2025 Jan 21;12:e66925. doi: 10.2196/66925 (PMC11795159; doi:10.2196/66925)
Supplement: Multimedia Appendix 1 [file mental_v12i1e66925_app1.docx]

**Online Questionnaires**

**For each question, please circle one that applies most to you**

| **Please specify your occupation:** | | | |
| --- | --- | --- | --- |
| o Allied Health | o Nursing | o Physician |  |

**Please enter Study ID:**

**_________**

**Attitudes towards people with schizophrenia**

| 1. For most people with schizophrenia, it is their own fault that they have schizophrenia. | | | | | | | | |
| --- | --- | --- | --- | --- | --- | --- | --- | --- |
| o 1 strongly disagree | o | o | o | o | o | o | o | o 9 strongly agree |
|  |  |  |  |  |  |  |  |  |
| 2. Most people with schizophrenia could have avoided contracting the condition. | | | | | | | | |
| o 1 strongly disagree | o | o | o | o | o | o | o | o 9 strongly agree |
|  |  |  |  |  |  |  |  |  |
| 3. How much do you personally care about the plight of people with schizophrenia? | | | | | | | | |
| o 1  Not at all | o | o | o | o | o | o | o | o 9  very much |
|  |  |  |  |  |  |  |  |  |
| 4. Our society does not do enough to help people with schizophrenia. | | | | | | | | |
| o 1 strongly disagree | o | o | o | o | o | o | o | o 9 strongly agree |
|  |  |  |  |  |  |  |  |  |
| 5. Compared with other social problems we face today (e.g., crime, education, drugs, homelessness, environmental protection, energy conservation), how would you rate the importance of helping people with schizophrenia? | | | | | | | | |
| o 1  not at all important | o | o | o | o | o | o | o | o 9 extremely important |
|  |  |  |  |  |  |  |  |  |
| 6. Our society should do more to protect the welfare of people with schizophrenia. | | | | | | | | |
| o 1 strongly disagree | o | o | o | o | o | o | o | o 9 strongly agree |
|  |  |  |  |  |  |  |  |  |
| 7. In general, what are your feelings toward people with schizophrenia? | | | | | | | | |
| o 1 strongly negative | o | o | o | o | o | o | o | o 9 extremely positive |

**Desire for Social Distance Scale**

| 1. How willing would you be to move next door to someone with schizophrenia? | | | | | |
| --- | --- | --- | --- | --- | --- |
|  | o 0  Yes, definitely | o 1  Yes, probably | o 2  Probably not | o 3  Definitely not |  |
| 2. How willing would you be to spend an evening with someone with schizophrenia? | | | | | |
|  | o 0  Yes, definitely | o 1  Yes, probably | o 2  Probably not | o 3  Definitely not |  |
| 3. How willing would you be to make friends with someone with schizophrenia? | | | | | |
|  | o 0  Yes, definitely | o 1  Yes, probably | o 2  Probably not | o 3  Definitely not |  |
| 4. How willing would you be to have someone with schizophrenia start working closely with you on a job? | | | | | |
|  | o 0  Yes, definitely | o 1  Yes, probably | o 2  Probably not | o 3  Definitely not |  |
| 5. How willing would you be to have someone with schizophrenia marry into your family? | | | | | |
|  | o 0  Yes, definitely | o 1  Yes, probably | o 2  Probably not | o 3  Definitely not |  |
| 6. How willing would you be to have someone with schizophrenia as a citizen of your country? | | | | | |
|  | o 0  Yes, definitely | o 1  Yes, probably | o 2  Probably not | o 3  Definitely not |  |
| 7. How willing would you be to have someone with schizophrenia visit your country as a non-citizen? | | | | | |
|  | o 0  Yes, definitely | o 1  Yes, probably | o 2  Probably not | o 3  Definitely not |  |
| 8. How willing would you be to have someone with schizophrenia be excluded from associating with your country in  any way? | | | | | |
|  | o 0  Yes, definitely | o 1  Yes, probably | o 2  Probably not | o 3  Definitely not |  |

**Personal Stigma Scale**

| 1. People with schizophrenia could snap out of it if they wanted. | | | | | |
| --- | --- | --- | --- | --- | --- |
|  | o 0  Strongly disagree | o 1  Disagree | o 2  Neither agree nor disagree | o 3  Agree | o 4  Strongly agree |
| 2. Schizophrenia is a sign of personal weakness. | | | | | |
|  | o 0  Strongly disagree | o 1  Disagree | o 2  Neither agree nor disagree | o 3  Agree | o 4  Strongly agree |
| 3. Schizophrenia is not a real medical illness. | | | | | |
|  | o 0  Strongly disagree | o 1  Disagree | o 2  Neither agree nor disagree | o 3  Agree | o 4  Strongly agree |
| 4. People with schizophrenia are dangerous. | | | | | |
|  | o 0  Strongly disagree | o 1  Disagree | o 2  Neither agree nor disagree | o 3  Agree | o 4  Strongly agree |
| 5. It is best to avoid people with schizophrenia so that you don’t become schizophrenic yourself. | | | | | |
|  | o 0  Strongly disagree | o 1  Disagree | o 2  Neither agree nor disagree | o 3  Agree | o 4  Strongly agree |
| 6. People with schizophrenia are unpredictable. | | | | | |
|  | o 0  Strongly disagree | o 1  Disagree | o 2  Neither agree nor disagree | o 3  Agree | o 4  Strongly agree |
| 7. If I had schizophrenia I would not tell anyone. | | | | | |
|  | o 0  Strongly disagree | o 1  Disagree | o 2  Neither agree nor disagree | o 3  Agree | o 4  Strongly agree |
| 8. I would not employ someone if I knew they had experienced schizophrenia. | | | | | |
|  | o 0  Strongly disagree | o 1  Disagree | o 2  Neither agree nor disagree | o 3  Agree | o 4  Strongly agree |
| 9. I would not vote for a politician if I knew they had experienced schizophrenia. | | | | | |
|  | o 0  Strongly disagree | o 1  Disagree | o 2  Neither agree nor disagree | o 3  Agree | o 4  Strongly agree |

**Interpersonal Reactivity Index, Empathetic Concern Subscale**

| 1. I often have tender, concerned feelings for people less fortunate than me. | | | | | | | | |
| --- | --- | --- | --- | --- | --- | --- | --- | --- |
|  | o 0  Does not describe me well | o 1 | o 2 | | o 3 | | o 4  Describes me very well | |
| 2. Sometimes I don't feel very sorry for other people when they are having problems. | | | | | | | | |
|  | o 0  Does not describe me well | o 1 | o 2 | | o 3 | | o 4  Describes me very well | |
| 3. When I see someone being taken advantage of, I feel kind of protective towards them. | | | | | | | | |
|  | o 0  Does not describe me well | o 1 | o 2 | | o 3 | | o 4  Describes me very well | |
| 4. Other people's misfortunes do not usually disturb me a great deal. | | | | | | | | |
|  | o 0  Does not describe me well | o 1 | o 2 | | o 3 | | o 4  Describes me very well | |
| 5. When I see someone being treated unfairly, I sometimes don't feel very much pity for  them. | | | | | | | | |
|  | o 0  Does not describe me well | o 1 | o 2 | | o 3 | | o 4  Describes me very well | |
| 6. I am often quite touched by things that I see happen. | | | | | | | | |
|  | o 0  Does not describe me well | o 1 | o 2 | | o 3 | | o 4  Describes me very well | |
| 7. I would describe myself as a pretty soft-hearted person. | | | | | | | | |
|  | o 0  Does not describe me well | o 1 | o 2 | | o 3 | | o 4  Describes me very well | |
|  |  |  | |  | |  | |  |

**Demographic data (for both groups at baseline only)**

**Please tick the box that best describes you**

| 1. Please specify your age | | | | | | | | | | | | | | | | | | | | | | | | | | | | | | | | | | | | | | | |
| --- | --- | --- | --- | --- | --- | --- | --- | --- | --- | --- | --- | --- | --- | --- | --- | --- | --- | --- | --- | --- | --- | --- | --- | --- | --- | --- | --- | --- | --- | --- | --- | --- | --- | --- | --- | --- | --- | --- | --- |
|  | | _________ | | | |  | |  | | | | |  | | | | |  |  |  |  |  |  |  |  |  |  |  |  |  |  |  |  |  |  |  |  |  |  |
| 2. Please specify your gender | | | | | | | | | | | | | | | | | | | | | | | | | | | | | | | | | | | | | | | |
|  | | o Male | | o Female | | | | | |  | | | | | | |  | | | | |  | | | | | |  | | |  | | | | |  | | | |
| 3. Please specify your ethnicity | | | | | | | | | | | | | | | | | | | | | | | | | | | | | | | | | | | | | | | |
|  | | o Chinese | o Malay | | | | o Indian | | | | | | | o Eurasian | | | | | | **o Others:**  _______ | | | | | | | |  | | |  | | | | |  | | | |
| 4. Please specify your marital status | | | | | | | | | | | | | | | | | | | | | | | | | | | | | | | | | | | | | | | |
|  | | o Single, never married | | | | | | | o Attached | | | | | | | o Engaged | | | | | | | o Married | | | | o Separated | | | | | o Divorced | | | | | | |  |
| o Widowed | | | | | | | | | | | | | | | | | | | | | | | | | | | | | | | | | | | | | | | |
| 5. Please specify your religion: | | | | | | | | | | | | | | | | | | | | | | | | | | | | | | | | | | | | | | | |
|  | | o Christian | | o Catholic | | | | | | | o Buddhist | | | | | | o Taoism | | | | | o Muslim | | | | | |  | | |  | | | | | |  | | |
|  | | o Hindu | | O Free thinker | | | | | | | **o Others:** | | | | | |  | | | | |  | | | | | |  | | |  | | | | | |  | | |
| 6. Please specify your highest degree completed. | | | | | | | | | | | | | | | | | | | | | | | | | | | | | | | | | | | | | | | |
|  | o ‘N’ levels | | | o ‘O’ levels | | | | | | o Nitec | | | | | o Diploma | | | | | | o Degree | | | | o Masters | | | | | o PhD | | | |  | | | | | |
|  | | | | | | | | | | | | | | | | | | | | | | | | | | | | | | | | | |  | | | | | |
| 7. Please specify your occupation | | | | | | | | | | | | | | | | | | | | | | | | | | | | | | | | | |  | | | | | |
|  | o Psychiatrist | | | | o Family physician | | | | | | | o Resident | | | | | | | o Medical Officer | | | | | | | o Dentist | | | |  | | | | | | | | | |
|  | o Nurse | | | | o Medical Social worker o Case manager | | | | | | | | | | | | | | | | | | | | o OT | | | | O PT | | | | | | **o Others:** | | | | |
| 8. Years of service at IMH (exclude years of study) | | | | | | | | | | | | | | | | | | | | | | | | | | | | | | | | | | | | | | | |
|  | | o < 1 year | | o 1-3 years | | | | | | o 4-5 years | | | | | | | o 6-9 years | | | | | | | o 10-15 years | | | | | | | | |  | | | | |  |  |
|  | | o 16-20 years | | o 21-24 years | | | | | | o 25-30 years | | | | | | | o > 31 years | | | | | | |  | | | | | | | | |  | | | | |  |  |
| 9. Years of service in mental health sector, including IMH (exclude years of study) | | | | | | | | | | | | | | | | | | | | | | | | | | | | | | | | | | | | | | | |
|  | | o < 1 year | | o 1-3 years | | | | | | o 4-5 years | | | | | | | o 6-9 years | | | | | | |  |  |  |  |  |  |  |  |  |  |  |  |  |  |  |  |
|  | | o 16-20 years | | o 21-24 years | | | | | | o 25-30 years | | | | | | | o > 31 years | | | | | | |  |  |  |  |  |  |  |  |  |  |  |  |  |  |  |  |

10. Do you have a close friend or family with a mental health condition?

| o Yes | o No |
| --- | --- |

**User satisfaction questionnaire *(Post-intervention only, both groups)***

| \| 1. I find the intervention engaging. \| \| \| \| \| \| \| \| --- \| --- \| --- \| --- \| --- \| --- \| --- \| \| o 1  Strongly Disagree \| o 2  Disagree \| o 3  Slightly Disagree \| o 4  Neither Agree  or Disagree \| o 5  Slightly Agree \| o 6  Agree \| o 7  Strongly agree \| \| 2. The VR simulation looks realistic. \| \| \| \| \| \| \| \| o 1  Strongly Disagree \| o 2  Disagree \| o 3  Slightly Disagree \| o 4  Neither Agree  or Disagree \| o 5  Slightly Agree \| o 6  Agree \| o 7  Strongly agree \| \| 3. The intervention helps me to better understand psychotic experiences like auditory hallucination. \| \| \| \| \| \| \| \| o 1  Strongly Disagree \| o 2  Disagree \| o 3  Slightly Disagree \| o 4  Neither Agree  or Disagree \| o 5  Slightly Agree \| o 6  Agree \| o 7  Strongly agree \| \| 4. The intervention helps me to understand what people with schizophrenia experience and feel. \| \| \| \| \| \| \| \| o 1  Strongly Disagree \| o 2  Disagree \| o 3  Slightly Disagree \| o 4  Neither Agree  or Disagree \| o 5  Slightly Agree \| o 6  Agree \| o 7  Strongly agree \| \| 5. The intervention helps me to improve my care towards people with schizophrenia. \| \| \| \| \| \| \| \| o 1  Strongly Disagree \| o 2  Disagree \| o 3  Slightly Disagree \| o 4  Neither Agree  or Disagree \| o 5  Slightly Agree \| o 6  Agree \| o 7  Strongly agree \| \| 6. This virtual reality intervention is effective in educating about psychotic experiences like auditory hallucination. \| \| \| \| \| \| \| \| o 1  Strongly Disagree \| o 2  Disagree \| o 3  Slightly Disagree \| o 4  Neither Agree  or Disagree \| o 5  Slightly Agree \| o 6  Agree \| o 7  Strongly agree \| | | | | | | | | | |
| --- | --- | --- | --- | --- | --- | --- | --- | --- | --- | --- | --- | --- | --- | --- | --- | --- | --- | --- | --- | --- | --- | --- | --- | --- | --- | --- | --- | --- | --- | --- | --- | --- | --- | --- | --- | --- | --- | --- | --- | --- | --- | --- | --- | --- | --- | --- | --- | --- | --- | --- | --- | --- | --- | --- | --- | --- | --- | --- | --- | --- | --- | --- | --- | --- | --- | --- | --- | --- | --- | --- | --- | --- | --- | --- | --- | --- | --- | --- | --- | --- | --- | --- | --- | --- | --- | --- | --- | --- | --- | --- | --- | --- | --- |
| 7. Please list the strengths of the intervention. | | | | | | | | | |
| ___________________________________ | | | | | | | | | |
| 8. Please list how the intervention can be improved. | | | | | | | | | |
| ___________________________________ | | | | | | | | | |
| 9. What did you enjoy most about the VR intervention?  ____________________________________ | | | | | | | | | |
|  | | | | | | | | | |
| 10. What did you enjoy least about the VR intervention?  ____________________________________ | | | | | | | | | |
| **User sati Visually induced motion sickness susceptibility questionnaire *(Post-intervention only, both groups)*** | | | | | | | | | |
| How often have you experienced each of the following symptoms when reviewing the VR intervention?   1. Nausea | | | | | | | | |  |
|  | o 0  Never | O 1  Rarely | o 2  Sometimes | o 3  Often |  |  |  |  |  |
|  | 2. Headache |  |  |  |  |  |  |  |  |
|  | o 0  Never | O 1  Rarely | o 2  Sometimes | o 3  Often |  |  |  |  |  |
|  | 3. Dizziness |  |  |  |  |  |  |  |  |
|  | o 0  Never | O 1  Rarely | o 2  Sometimes | o 3  Often |  |  |  |  |  |
|  | 4. Fatigue |  |  |  |  |  |  |  |  |
|  | o 0  Never | O 1  Rarely | o 2  Sometimes | o 3  Often |  |  |  |  |  |
|  | 5. Eye strain |  |  |  |  |  |  |  |  |
|  | o 0  Never | O 1  Rarely | o 2  Sometimes | o 3  Often |  |  |  |  |  |
|  | 6. Have any of these symptoms stopped you using any of these devices or made you avoid viewing the intervention? | | | | | | | |  |
|  | o 0  Never | O 1  Rarely | o 2  Sometimes | o 3  Often |  |  |  |  |  |
|  |  |  |  |  |  |  |  |  |  |
